# Genetic- and culture-based tools for studying *Bacteroides fragilis*

**DOI:** 10.1128/mra.00006-25

**Published:** 2025-03-25

**Authors:** Matthew K. Schnizlein, Abhishek A. Dubey, Aretha Fiebig, Sean Crosson

**Affiliations:** 1 Department of Microbiology, Genetics and Immunology, Michigan State University 3078, East Lansing, Michigan, USA; 2 Leibniz Institute on Aging, Fritz Lipmann Institute 28407, Jena, Thuringia, Germany; Indiana University Bloomington, Bloomington, Indiana, USA

**Keywords:** *Bacteroides*, molecular genetics, metabolic modeling, anaerobes

## Abstract

The relatively limited availability of genetic tools has hampered mechanistic studies of *Bacteroides fragilis,* an opportunistic anaerobe that constitutes 1%–5% of the gut microbiota in healthy humans. Here we describe novel vectors for *B. fragilis* gene deletion and expression as well as a semi-defined media for cultivation of *B. fragilis* str. P207.

## ANNOUNCEMENT

Limited genetic tractability of newly isolated bacteria has hindered their study. Here we describe additional tools for genetic manipulation of the gut microbe *Bacteroides fragilis*, specifically the strain P207 which was isolated from an inflamed ileoanal pouch (1–5). In this announcement, we build on work by other researchers (6–13) and report the construction of a semi-defined growth medium as well as novel plasmids for mechanistic studies in *B. fragilis*, which include vectors for constitutive and inducible expression that have versatile multiple cloning sites as well as options for C-terminal 3xFLAG tagging and/or N- and C-terminal fluorescent protein fusion.

### Genetic-based tools

**Fig 1:**
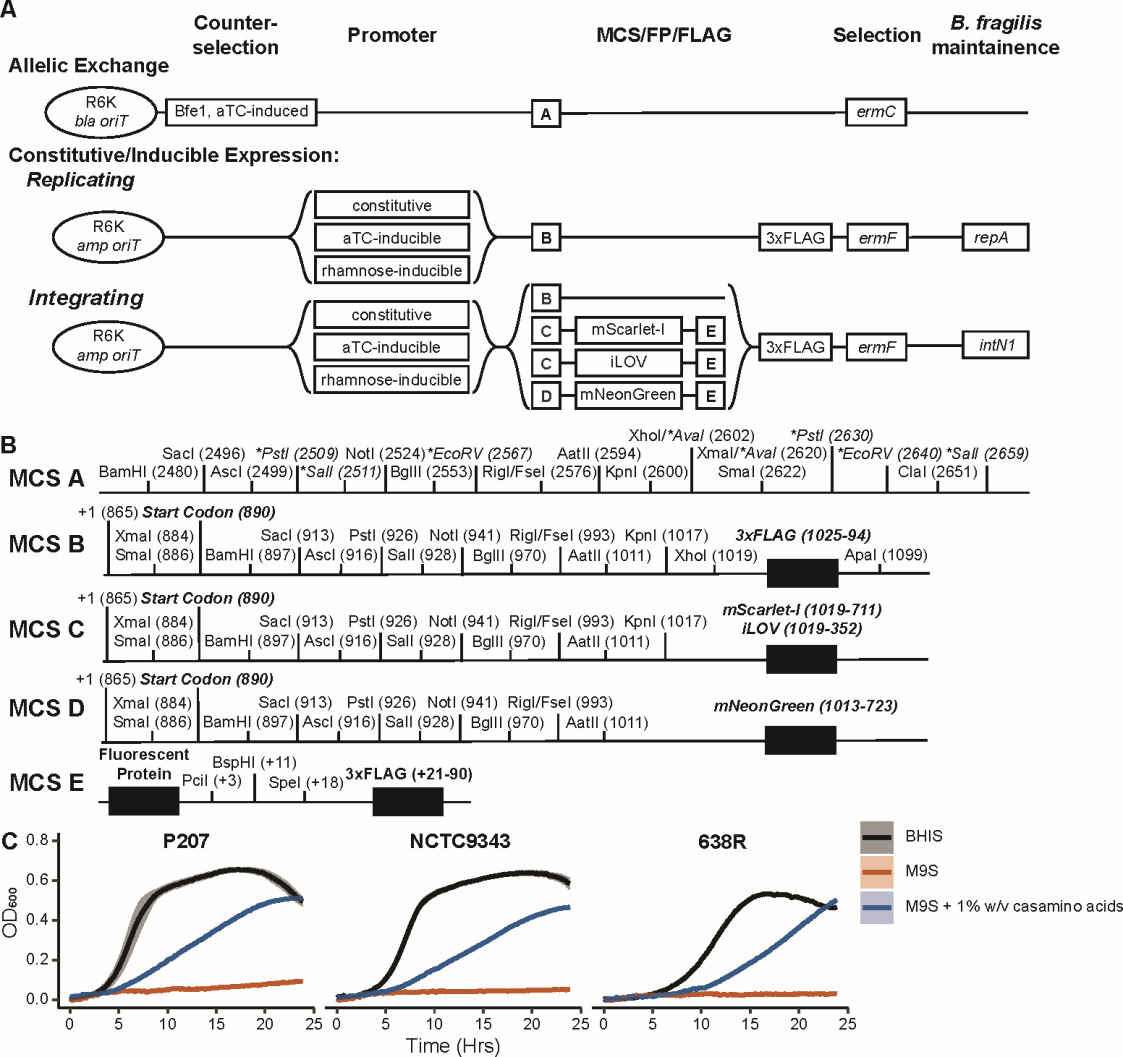
(A) A graphical summary of allelic exchange, replicating and integrating vectors designed in this study. (B) A representation of multiple cloning sites A–E. An asterisk/italic font indicates that the site appears more than once in the MCS. (C) OD_600_ growth measurements of *B. fragilis* strains P207, NCTC9343, and 638R grown in BHIS, M9S, or M9S + 1% (wt/vol) casamino acids.

We enhanced the multiple cloning cassette (mcs) of the allelic exchange vector created by García-Bayona et al. (Fig. 1A and B) using a derivation of the mcs regions in the pCOLADuet1 vector (Novagen, Millipore Sigma) (6). We also generated a series of replicating (RepA) and integrating vectors (IntN1; Fig. S1B through D) (14, 15). Each vector series has optional C-terminal 3xFLAG tag fusion (Table 1) as well as options for N- and C-terminal fusion with the fluorescent proteins mScarlet-I, mNeonGreen, and iLOV. Since *B. fragilis* is an anaerobic organism, fluorophores such as mNeonGreen and mScarlet-I require aerobic recovery to stimulate maturation (16). While we had some success with obtaining mNeonGreen fluorescence after 1 hour of aerobic recovery, mScarlet-I, which has a longer maturation time, requires further troubleshooting. The anaerobic fluorescent protein iLOV also requires optimization due to low fluorescent signal.

**TABLE 1:** Vectors and sequencing primers

Vector ID	Maintenance	Promoter	Selection	Features	AddGene ID
pMS01	Allelic exchange	no inserted promoter	*ermC*	aTC-inducible bfe1 counterselection	230064
pMS02	Integrating	Constitutive	*ermF*	C-terminal 3x-FLAG	230066
pMS03	Replicating				230937
pMS04	Integrating	aTC-inducible			230068
pMS05	Replicating				230069
pMS06	Integrating	Rhamnose-inducible			230070
pMS07	Replicating				230071
pMS08	Integrating	Constitutive		N- or C-terminal fusion with mScarletI; C-terminal 3x-FLAG	230072
pMS09	Integrating	Constitutive		N- or C-terminal fusion with iLOV; C-terminal 3x-FLAG	230073
pMS10	Integrating	Constitutive		N- or C-terminal fusion with mNeonGreen; C-terminal 3x-FLAG	230074

### Culture-based tools

Due to poor growth of *B. fragilis* str. P207 in an established defined medium (i.e., M9S; see supplemental methods), we optimized growth media composition by predicting auxotrophies using the fba_tools application in KBase (17–19). This identified putative auxotrophies for siroheme, L-valine, and putrescine. We also found predicted auxotrophies for *B. fragilis* strains NCTC9343 and 638R which included L-valine as well as other amino acids. Since supplementing with single branched-chain amino acids suppresses growth (20), we supplemented M9S with 1% casamino acids, which significantly improved culture density across all strains (Fig. 1C).

We provide primer sequences to substitute antibiotic resistance genes as well as expression promoters, if others wish to expand upon these vectors. The fluorescent proteins can also be transferred into any of the other promoter vectors using pre-designed primers. As fluorescent protein use continues to be an ongoing challenge in anaerobic organisms, we hope that these vectors will facilitate the further optimization needed.

## Data Availability

You can access the vectors and their sequences via AddGene (#230064-230066, 230068-230074, and 230937). In addition, you can find vector maps and sequences, primers (Table S1), and methods on GitHub (https://github.com/mschnizlein/bfrag_genetictools).

## AFTER EPUB

*[This article was published on 25 March 2025. An Addendum with supporting references was included in the current version, posted on 21 April 2025.]*

## ADDENDUM

Although we were unable to cultivate *B. fragilis strains*, including P207, in a defined medium lacking amino acids, prior studies have demonstrated that *B. fragilis* can grow under fully anaerobic conditions in media supplemented with cysteine but without additional amino acids (6, 21–24). *B. fragilis* has also been cultivated in anaerobic defined media supplemented only with methionine and cysteine (25). We expect that strain P207 is similarly capable of growth under these conditions. However, in our experiments, cultures were exposed to oxygen during preparation and only transferred to the anaerobic chamber after inoculation. This likely impaired growth by oxidizing dihydroxyacid dehydratase (23), which is required for branched chain amino acid biosynthesis.

## References

[1] Wexler HM. 2007. Bacteroides: the good, the bad, and the nitty-gritty. Clin Microbiol Rev 20:593–621. doi:10.1128/CMR.00008-07 17934076 PMC2176045

[2] Patrick S. 2022. A tale of two habitats: Bacteroides fragilis, a lethal pathogen and resident in the human gastrointestinal microbiome. Microbiology (Reading) 168. doi:10.1099/mic.0.001156 35404220

[3] Vineis JH, Ringus DL, Morrison HG, Delmont TO, Dalal S, Raffals LH, Antonopoulos DA, Rubin DT, Eren AM, Chang EB, Sogin ML. 2016. Patient-specific Bacteroides genome variants in pouchitis. MBio 7:e01713-16. doi:10.1128/mBio.01713-16 27935837 PMC5111406

[4] Mullowney MW, Fiebig A, Schnizlein MK, McMillin M, Rose AR, Koval J, Rubin D, Dalal S, Sogin ML, Chang EB, Sidebottom AM, Crosson S. 2024. Microbially catalyzed conjugation of GABA and tyramine to bile acids. J Bacteriol 206:e0042623. doi:10.1128/jb.00426-23 38174933 PMC10810215

[5] Fiebig A, Schnizlein MK, Pena-Rivera S, Trigodet F, Dubey AA, Hennessy MK, Basu A, Pott S, Dalal S, Rubin D, Sogin ML, Eren AM, Chang EB, Crosson S. 2024. Bile acid fitness determinants of a Bacteroides fragilis isolate from a human pouchitis patient. MBio 15:e0283023. doi:10.1128/mbio.02830-23 38063424 PMC10790697

[6] García-Bayona L, Comstock LE. 2019. Streamlined genetic manipulation of diverse Bacteroides and Parabacteroides isolates from the human gut microbiota. MBio 10:e01762-19. doi:10.1128/mBio.01762-19 31409684 PMC6692515

[7] Lim B, Zimmermann M, Barry NA, Goodman AL. 2017. Engineered regulatory systems modulate gene expression of human commensals in the gut. Cell 169:547–558. doi:10.1016/j.cell.2017.03.045 28431252 PMC5532740

[8] Hamady ZZR, Farrar MD, Whitehead TR, Holland KT, Lodge JPA, Carding SR. 2008. Identification and use of the putative Bacteroides ovatus xylanase promoter for the inducible production of recombinant human proteins. Microbiology (Reading) 154:3165–3174. doi:10.1099/mic.0.2008/019109-0 18832322

[9] Horn N, Carvalho AL, Overweg K, Wegmann U, Carding SR, Stentz R. 2016. A novel tightly regulated gene expression system for the human intestinal symbiont Bacteroides thetaiotaomicron. Front Microbiol 7:1080. doi:10.3389/fmicb.2016.01080 27468280 PMC4942465

[10] Parker AC, Jeffrey Smith C. 2012. Development of an IPTG inducible expression vector adapted for Bacteroides fragilis. Plasmid 68:86–92. doi:10.1016/j.plasmid.2012.03.002 22487080 PMC3389198

[11] Mimee M, Tucker AC, Voigt CA, Lu TK. 2015. Programming a human commensal bacterium, Bacteroides thetaiotaomicron, to sense and respond to stimuli in the murine gut microbiota. Cell Syst 1:62–71. doi:10.1016/j.cels.2015.06.001 26918244 PMC4762051

[12] Orth P, Schnappinger D, Hillen W, Saenger W, Hinrichs W. 2000. Structural basis of gene regulation by the tetracycline inducible Tet repressor-operator system. Nat Struct Biol 7:215–219. doi:10.1038/73324 10700280

[13] Lederer T, Takahashi M, Hillen W. 1995. Thermodynamic analysis of tetracycline-mediated induction of Tet repressor by a quantitative methylation protection assay. Anal Biochem 232:190–196. doi:10.1006/abio.1995.0006 8747474

[14] Rajeev L, Segall A, Gardner J. 2007. The Bacteroides NBU1 integrase performs a homology-independent strand exchange to form a holliday junction intermediate. J Biol Chem 282:31228–31237. doi:10.1074/jbc.M705370200 17766246

[15] Betteridge T, Yang J, Pittard AJ, Praszkier J. 2003. Interaction of the initiator protein of an IncB plasmid with its origin of DNA replication. J Bacteriol 185:2210–2218. doi:10.1128/JB.185.7.2210-2218.2003 12644491 PMC151506

[16] Zhang C, Xing X-H, Lou K. 2005. Rapid detection of a gfp-marked Enterobacter aerogenes under anaerobic conditions by aerobic fluorescence recovery. FEMS Microbiol Lett 249:211–218. doi:10.1016/j.femsle.2005.05.051 16006057

[17] Allen B, Drake M, Harris N, Sullivan T. 2017. Using KBase to assemble and annotate prokaryotic genomes. Curr Protoc Microbiol 46:1E. doi:10.1002/cpmc.37 28800158

[18] Arkin AP, Cottingham RW, Henry CS, Harris NL, Stevens RL, Maslov S, Dehal P, Ware D, Perez F, Canon S, et al.. 2018. KBase: the United States department of energy systems biology knowledgebase. Nat Biotechnol 36:566–569. doi:10.1038/nbt.4163 29979655 PMC6870991

[19] Allen BH, Gupta N, Edirisinghe JN, Faria JP, Henry CS. 2022. Application of the metabolic modeling pipeline in KBase to categorize reactions, predict essential genes, and predict pathways in an isolate genome, p 291–320. In Navid A (ed), Microbial systems biology: methods and protocols. Springer US, New York, NY. doi:10.1007/978-1-0716-1585-0_13. 34719000

[20] Massey LK, Sokatch JR, Conrad RS. 1976. Branched-chain amino acid catabolism in bacteria. Bacteriol Rev 40:42–54. doi:10.1128/br.40.1.42-54.1976 773366 PMC413937

[21] Baughn AD, Malamy MH. 2002. A mitochondrial-like aconitase in the bacterium Bacteroides fragilis: implications for the evolution of the mitochondrial Krebs cycle. Proc Natl Acad Sci U S A 99:4662–4667. 11880608 10.1073/pnas.052710199 PMC123704

[22] García-Bayona L, Coyne MJ, Hantman N, Montero-Llopis P, Von SS, Ito T, Malamy MH, Basler M, Barquera B, Comstock LE. 2020. Nanaerobic growth enables direct visualization of dynamic cellular processes in human gut symbionts. Proc Natl Acad Sci U S A 117:24484–24493. 32938803 10.1073/pnas.2009556117 PMC7533675

[23] Meehan BM, Baughn AD, Gallegos R, Malamy MH. 2012. Inactivation of a single gene enables microaerobic growth of the obligate anaerobe Bacteroides fragilis. Proc Natl Acad Sci U S A 109:12153–12158. 22778399 10.1073/pnas.1203796109 PMC3409759

[24] Varel VH, Bryant MP. 1974. Nutritional features of Bacteroides fragilis subsp. fragilis. Appl Microbiol 28:251–257. 4853401 10.1128/am.28.2.251-257.1974 PMC186696

[25] Parker AC, Seals NL, Baccanale CL, Rocha ER. 2022. Analysis of Six tonB Gene Homologs in Bacteroides fragilis Revealed That tonB3 is Essential for Survival in Experimental Intestinal Colonization and Intra-Abdominal Infection Infect Immun 90:e0046921. 10.1128/IAI.00469-21 PMC8788773 34662212

